# The investigation of antibacterial activity of selected native plants from North of Iran

**Published:** 2015

**Authors:** H Koohsari, EA Ghaemi, M Sadegh Sheshpoli, M Jahedi, M Zahiri

**Affiliations:** * Department of Microbiology, Azadshahr Branch, Islamic Azad University, Azadshahr, Iran; ** Department of Microbiology, Golestan University of Medical Sciences, Gorgan, Iran; *** Department of Molecular Medicine, Golestan University of Medical Science, Gorgan, Iran; **** Young Researchers and Elite Club, Azadshahr Branch, Islamic Azad University, Azadshahr, Iran

**Keywords:** antibacterial effect, medicine plants, Disk Diffusion method, Well method, MBC

## Abstract

Plant derived products have been used for medicinal purposes during centuries. Bacterial resistance to currently used antibiotics has become a concern to public health. The development of bacterial super resistant strains has resulted in the currently used antibiotic agents failing to end many bacterial infections. For this reason, the search is ongoing for new antimicrobial agents, both by the design and by the synthesis of new agents, or through the search of natural sources for yet undiscovered antimicrobial agents. Herbal medications in particular have seen a revival of interest due to a perception that there is a lower incidence of adverse reactions to plant preparations compared to synthetic pharmaceuticals. Coupled with the reduced costs of plant preparations, this makes the search for natural therapeutics an attractive option. This research was carried out to assess the antibacterial activity aqueous and ethanolic extracts of six Azadshahr township Native plants in north of Iran against six species of pathogen bacteria by using three methods of Disk diffusion, Well method and MBC. The results of this research indicated that the effect of ethanol extracts were more than aqueous extract and among six plants, Lippia citriodora and Plantago major ethanol extract had the most antibacterial activity in any of the three methods. Gram-positive bacteria were more sensitive than gram-negative bacteria. Staphylococcus epidermidis and Staphylococcus aureus were the most susceptible Gram-positive bacteria.

## Introduction

Despite the apparent advantages of new medicine compared to traditional medicine, using chemical drugs represents a major drawback, which is becoming more threatening every day and causes antibiotic resistance. This is an acute problem that the World Health Organization has been confronted with. Thus, scientists are trying to find new antimicrobial drugs in a constant effort. Herbs used in the rich traditional medicine of Iran are a good source of finding such antimicrobial drugs. According to the estimation of WHO, 80% of the world’s population believe in the effects of herbal medication in treating diseases [**1**]. Organic plants produce secondary metabolites, which can be considered an important drug source having antibacterial and antifungal effects. In Iranian traditional medicine, the use of herbs is common in burns, skin disorders, infectious diseases, sepsis, and inflammation [**2**,**3**].

Considering the unique climate and great herbal drugs diversity of Golestan province, Azadshahr district was chosen to conduct the research. The object of the study was to evaluate the effects of aqueous extracts and ethanol extracts of 6 (six) endemic plants of Azadshahr district, namely Lippia citriodora, Plantago major, Sambucus ebulus, Althaea officinalis, Tilia bengonifolia, Adiantum capillus-veneris, on 6 (six) important human pathogenic bacteria including Escherichia coli, Staphylococcus aureus, Salmonella typhimurium, Staphylococcus epidermidis, Shigella dysentery and Enterococcus faecalis. In addition to different diseases, these Barcia also caused food poisoning.

## Materials and Methods

**Identification and collection of herbs**

Field operations were carried out to identify the natural habitat of herbs. After collecting different types by using the herbarium of Islamic Azad University of Gorgan, herbs were identified and confirmed. Then, the required parts (leaves, flowers, etc.) were cut and dried in an appropriate condition (dark and dry). After being dried, plants were grinded and their powder was used in extraction.

**Preparation of aqueous extract of herbs**

Sterile distilled water was used as a solvent in preparing the aqueous extracts. To prepare the aqueous extract, 30g of the powder was chosen and 100 cc of distilled water having a temperature of 70-80°C was added to an erlen containing the powder. Then it was covered with foil and put in a bain-marie-water bath- with a temperature of 60°C. After 24 hours, the erlen was removed from the water bath, and the mixture in the erlen was compressed, filtered with the filter paper and a Buchner funnel, and separated from the solvent by using the vacuum distillation method [**4**]. This extract was considered the pure extract and other concentrations were prepared with sterile distilled water.

**Preparing ethanol extracts of herbs**

In this study, ethanol of 70° and percolation method were used. Thus, 50g of herbal sample powder were added to a decanter and then 70° ethanol was assed stage by stage to it. In order to add ethanol, we heated it first and then poured it to the decanter. We continued to add ethanol until the herbs in the decanter were soaked completely and some ethanol was on the surface of the sample. After an hour, we turned on the decanter’s faucet so the solvent exited and then we returned it to the decanter and repeated the process for three times. The complete extraction, depending on the type of the organ (fruit, stem, root, leaves, and flower), required 24-72 hours. After the extraction, the solvent (ethanol) was separated from the extract by using rotary and the method of vacuum distillation [**4**]. This extract was considered pure (1000 mg/ ml) and, propylene glycol was used in order to obtain different concentrations.

When extracts were obtained, disk diffusion was used together with an MBC (minimum bactericidal concentration) method to determine the effects of antibacterial.

**Strains of bacteria**

The bacteria used in this study were Escherichia coli bacteria (PTCC 1399), Salmonella typhimurium (PTCC 1596), Shigella dysentery (PTCC 1188), Staphylococcus aureus (PTCC 1436), Staphylococcus epidermidis (PTCC 1435), and vancomycin-resistant Enterococcus faecalis (Van R 181). These bacteria were collected from the scientific and Industrial Research Organization of Iran

**Disk diffusion method**

In this method, blank disks manufactured by Padtan Teb Company were put in tubes containing dilutions of extracts and after 5 to 10 minutes extracts were absorbed to disks, incubated at a temperature of 37°C and dried completely and got ready for disks [**4**].

A microbial suspension of 0.5 McFarland (1.5 ×108CFU/ ml) was obtained from all the bacterial strains and then, surface culture was carried out by using swab on Mueller-Hinton agar plate. Next, disks containing different dilutions of extract were put on the surface of the culture with an appropriate distance from each other and from the edge of the plate. Plates were incubated for 24 hours at a temperature of 37°C and the results of antibacterial effect were calculated by measuring the inhibition zone diameter. To ensure, the test was repeated for each strain of bacteria and the mean of inhibition zone diameter in two times of repetition was chosen as the ultimate diameter [**5**]. The inhibition zone diameter was considered resistant when it was less than 7 mm, relatively resistant when it was 7-9 mm, relatively sensitive when it was 10-12 mm, and sensitive when it was more than 12 mm [**6**].

**Well method**

In the well method, a microbial suspension of 0.5 McFarland was put on the surface culture of Mueller-Hinton agar plate. Then, by means of a cork borer, some wells were drilled, having a 7mm diameter and 100 microliters of different concentrations of extracts were poured in the wells and were incubated for 24 hours at 37°C. After this period, the sensitivity, or resistance of bacteria were calculated by measuring the inhibition zone diameter around the wells [**7**].

**MBC method (Maximum Bacteriocidal Concentration)**

In order to determine the minimum bacteriocidal concentration, 100 microliters of different dillutions of extractions wwere put in ELISA microplate wells in the vicinity of 100 microliters suspension of every bacteria with a concentration of 106 CFU/ ml. After 24 hours of incubation at 37°C, all dillutions were put in Mueller-Hinton agar plate, then again the incubation of 24 hours at 37°C was repeated for the second time. Then, the minimum bacteriocidal concentration of extracts was determined by examining wheather the colony formation has been conducted or not [**8**].

## Results

Considering the ethanol extracts of the examined herbs, the anti microbial effects of Plantago major’s ethanol extraction was more than the other herbs. These extracts were effective even in 62.5 mg/ ml concentrations of Staphylococcus aureus and Staphylococcus epidermidis. The inhibition zone diameter for these two bacteria in the concentration of 62.5 mg/ ml is 18 and 17 respectively (**Table 1**). These results were also confirmed in the method of disk diffusion (**Table 2**). In MBC method, Staphylococcus epidermidis showed a remarkable sensitivity. In other words, the minimum bactericidal concentration of Plantago major for this bacterium was 15.62 mg/ ml (**Table 3**).

Ethanol extraction of Lippia citriodora as well as Plantago major showed a remarkable antibactorial effect; in the Well method and in the concentration of 500mg to ml, the inhibition zone diameter for Staphylococcus epidermidis, Staphylococcus aureus and Enterococcus faecalis was 26,30, and 12 mm respectively (**Table 1**).

Experiments carried on the aqueous extracts of the herbs, showed no significant antibacterial effect. However, the aqueous extracts of Sambucus ebulus against Staphylococcus aureus in the Well method and in the concentration of 500mg to ml showed an inhibition zone diameter of 14 mm which was not seen in other bacteria.

**Table 1:** Antibacterial activity of 6 herbs against the bacteria using the Well method

													Herb											
	Althaea officinalis				Sambucus ebulus				Tilia bengonifolia				A.capillus-veneris				Lippia citriodora				Plantago major			
Bacteria concentration \ mg/ml	62.5	125	250	500	62.5	125	250	500	62.5	125	250	500	62.5	125	250	500	62.5	125	250	500	62.5	125	250	500
Escherichia coli	0	0	0	9	0	0	0	0	0	0	0	0	0	0	0	0	0	0	0	9	0	0	0	0*
Staphylococcus aureus	0	10	14	19	14	20	22	24	0	0	0	12	0	0	11	15	15	19	24	26	18	26	30	33
Enterococcus faecalis	0	0	10	13	0	0	0	0	0	0	0	0	0	0	0	0	0	0	9	12	0	0	0	8
Shigella dysentery	0	0	0	9	0	0	0	0	0	0	0	0	0	0	0	0	0	0	0	0	0	0	0	9
Salmonella typhimurium	0	0	0	0	0	0	0	0	0	0	0	0	0	0	0	0	0	0	0	0	0	0	0	9
Staphylococcus epidermidis	0	12	15	20	14	17	21	25	0	10	12	15	0	0	12	16	18	24	28	30	17	23	27	30
* Inhibition zone diameter (mm)																								

**Table 2:** Antibacterial activity of 6 herbs against the bacteria using Disk diffusion

													Herb											
	Althaea officinalis				Sambucus ebulus				Tilia bengonifolia				A.capillus-veneris				Lippia citriodora				Plantago major			
Bacteria concentration \ mg/ml	62.5	125	250	500	62.5	125	250	500	62.5	125	250	500	62.5	125	250	500	62.5	125	250	500	62.5	125	250	500
Escherichia coli	0	0	0	0	0	0	0	0	0	0	0	0	0	0	0	0	0	0	0	0	0	0	0	*0
Staphylococcus aureus	0	0	0	17	10	12	17	21	0	0	0	9	0	0	0	0	11	15	20	21	17	23	27	29
Enterococcus faecalis	0	0	0	0	0	0	0	0	0	0	0	0	0	0	0	0	0	0	0	9	0	0	0	0
Shigella dysentery	0	0	0	0	0	0	0	0	0	0	0	0	0	0	0	0	0	0	0	0	0	0	0	0
Salmonella typhimurium	0	0	0	0	0	0	0	0	0	0	0	0	0	0	0	0	0	0	0	0	0	0	0	0
Staphylococcus epidermidis	0	0	10	13	10	13	17	23	0	0	0	14	0	0	0	0	13	16	23	26	14	20	24	26
* Inhibition zone diameter (mm)																								

**Table 3:** Minimum bactericidal concentration of 6 herb’s ethanol extraction against the bacteria

Bacteria \ Herb	Althaea officinalis	Sambucus ebulus	Tilia bengon.	A.capillus-veneris	Lippia citriodora	Plantago major
Escherichia coli	125	125	250	250	250	125*
S. aureus	31/25	62/5	125	125	62/5	31/25
E. faecalis	125	125	250	250	250	125
S. dysentery	250	125	250	125	250	125
S. typhimurium	62/5	125	250	250	125	125
S. epidermidis	31/25	62/5	62/5	62/5	15/62	15/62
* minimum bactericidal concentration (MBC)): mg/ ml)						

Statistically compared, the most antibacterial effects belong to the ethanol extraction of Plantago major and then Lippia citriodora and the least antibacterial effects belong to Tilia bengonifolia and Adiantum capillus-veneris. The differences between these these six herbs were statistically significant (P<0.001) (**Table 4**).

**Table 4:** The comparison of inhibitory effect of herbs

			Value 1		
		Resistant	Intermediate	sensitive	Total
Extract	Adiantum capillus	91.7%	4.2%	4.2%	100.0%
	Althaea officinalis	72.9%	12.5%	14.6%	100.0%
	Lippia citriodora	58.3%	10.4%	31.3%	100.0%
	Plantago major	62.5%	4.2%	33.3%	100.0%
	Sambucus ebulus	66.7%	6.3%	27.1%	100.0%
	Tilia bengonifolia	87.5%	8.3%	4.2%	100.0%
Total		73.3%	7.6%	19.1%	100.0%
*P<0.001*						

Regardless of the type of plant, Staphylococcus aureus 52%, Staphylococcus epidermidis 59%, and Enterococcus faecalis 2.1% showed sensitivity in their ethanol extracts compared to other herbs. However, gram-negative bacteria demonstrated a high resistance against the ethanol extracts and their sensitivity was less than 1%. As shown in **Table 5** this difference was significant.

**Table 5:** Comparing the sensitivity of different bacteria against the herb’s extracts

			Value 1		
		Resistant	Intermediate	sensitive	Total
Bacteria	S. aureus	33.3%	14.6%	52.1%	100.0%
	S. epidermidis	28.6%	12.2%	59.2%	100.0%
	E. faecalis	89.4%	8.5%	2.1%	100.0%
	E. coli	95.8%	4.2%	.0%	100.0%
	S. typhimurium	97.9%	2.1%	.0%	100.0%
	S. dysenteries	95.8%	4.2%	.0%	100.0%
Total		73.3%	7.6%	19.1%	100.0%
*P<0.001*						

## Discussion

One of the results of this study is related to the antibacterial effects of Plantago major’s ethanol extraction. In a research that Sharifa et al. [**9**] conducted, antibacterial and anti fungal effects of ethanol and methanol extracts of Plantago major were stipulated. Also, their results showed that the aquaous extract of this herb has no antibacterial effect against gram-positive and gram-negative bacteria. Their results are in congruence with our result [**9**].

Razik and et al. studied the antibacterial effects of Plantago major and Ceratonia Siliqua. They used the Well method to conduct their research, their results showing that Plantago major has more antibacterial effects as compared to other herb, especially against gram-positive bactria such as Staphylococcus aureus. Staphylococcus aureus was one of the most sensitive bacteria and Escherichia coli and Enterococcus were the most resistant which are in line with our results [**10**].

Ethanol extraction of Lippia citriodora showed some remarkable anti bacterial effects. Also, Ansari et al. [**5**] examined the antibacterial effects of Lippia citriodora’s essential oil against Methicillin-resistant Staphylococcus aureus. They used disk diffusion and MIC methods [**5**].

According to the tests, sensitivity of Staphylococcus epidermidis and Staphylococcus aureus against Adiantum capillus-veneris’s ethanol extract was confirmed. These results were consistent with [**11**].

In different studies, the sensitivity of gram-positive bacteria was studied in relation to herbal extracts. For instance, I.E. Cock studied the antibacterial effects of 39 methanol extracts of 25 Australian herbs against two gram-positive bacteria of Bacillus cereus and Bacillus subtilis and two gram-negative bacteria of Pseudomonas aeruginosa and Aeromonas hydrophila. He used the Disk diffusion method. The results showed the sensitivity of gram-positive bacteria [**12**]. In addition, Bishnu [**8**] mentioned the antibacterial effects of diffent extracts of India and Nepal. 10 important human pathogenic bacteria was investigated and the Well method wad used. Extracts were more effective in gram-positive bacteria compared to gram-negative bacteria. The most sensitive bacteria were Bacillus subtilis and Staphylococcus aureus and the most resistant bacteria were E. coli, Shigella disenteriae, Klebsiella pneumoniae and Salmonella Typhimurium [**8**] .

Cynthia Walter et al. studied the methanol effects of 10 herbal drugs in Pakistan and concluded the sensitivity of gram-positive bacteria. They used the Well method [**7**] .

In fact, gram-positive bacteria are more sensitive to herbal extracts than gram-negative bacteria. This may be because of Inherent tolerance of gram negatives and the nature and composition of herbs. According to the studies, the cell walls of gram-positive bacteria compared with gram-negative bacteria, are more sensitive to many of anti-boutiques, antimicrobial chemical compounds [**13**] and even many herbal drugs [**9**]. Lipopolysaccharides layer and periplasmic space of gram-negative bacteria are the reasons of relative resistance of gram-negative bacteria. 

In our study, the Well method showed the anti-bacterial effect of herbal extracts more than the Disk diffusion method. In some similar studies like [**7**,**14**,**15**], the Well method demonstarated more inhibitory effects compared to the Disk diffusion method. This could be because of the fact that the influence of drugs on blank paper disks was less, so the release of drug molecules to the surface of the disk culture of bacteria was less than the influence of drugs from the Well method to the culture.

In fact, the effects of herbs depend on their secondary metabolites. Since the ecosystems and different conditions play an important role in the biosynthesis of secondary metabolites, the secondary metabolites are different. Environmental factors are so important in the production of secondary metabolites of herbal drugs. Factors such as temperature, precipitation, light intensity, and altitude, which determine the climate of a region, affect the accumulation of secondary metabolites [**16**]. It has been proved that the different levels of active ingredients in herbal drugs are affected by climate. However, the accumulation and distribution of secondary metabolites is not equal and the differences in results of studies may be because of the differences in climate effects of different variations of herbs.

As a result, natural products with herbal origin play an important role in treating diseases and in considering the increase in the antibiotic resistance in pathogenic bacteria, the search for antimicrobial drugs being of utmost importance. Also, the study of metabolism and mechanism of compounds of these herbs could lead to new and more effective drugs.

**Acknowledgments**

Special thanks to Islamic Azad University of Azadshahr that supported us financially during these projects. 

## References

[1] Capasso L (1998). 5300 years ago, the Ice Man used natural laxatives and antibiotics. Lancet.

[2] Oussalah M, Caillet S, Saucie L, Lacroix M (2007). Inhibitory effects of selected plant essential oils on the growth of four pathogenic bacteria: E. coli O157:H7, Salmonella Typhimurium, Staphylococcus aureus and Listeria monocytogenes. vol. 18.

[3] Shahidi B (2004). Evaluation of antibacterial properties of some medicinal plants used in Iran. Journal of Ethnopharmacology.

[4] MaRhhadian NV, Rakhshandeh H (2008). Antibacterial and antifungal effects of Nigella sativa extracts against S. Aureus, P Aeroginosa and C. Albicans. Pakistan Jornal Medical Sci.

[5] Ansari M, Tehrani MS, Larijani K (2012). Antibacterial activity of Lippa citriodora herb essence against MRSA Staphylococcus aureaus. African Journal of Microbiology Research.

[6] Dulger B, Gonuz A (2004). Antimicrobial Activity of Certain Plants used in Turkish Traditional Medicine. Asian Journal of Plant Sciences.

[7] Walter C, Shinvari ZK, Afzal I, Malik RN (2011). Antibacterial Activity in Herbal Products Used in Pakistan. Pakistan Journal Of Botany.

[8] Joshi B, Lekhak S, Shorma A (2009). Antibacterial Property of Different Medicinal Plants: Ocimum sanctum, Cinnamomum zeylanicum, Xanthoxylum armatum and Origanum majorana. Nepal Journals Onliney.

[9] Sharifa AA, Neoh YL, Iswadi MI, Khairul O, Abdul Halim M, Jamalubin M, Mohamed Azman AB, Hing HL (2008). Effects of Methanol, Ethanol and Aqueous Extract of Plantago majoron Gram Positive Bacteria. Gram Negative Bacteria and Yeast, Annals of Microscopy.

[10] Abd Razik BM, Hasan HA, Murtadha MK (2012). The Study of Antibacterial Activity of Plantago Major and Ceratonia Siliqua. The Iraqi Postgraduate.

[11] Mahboubi A, Kamalinejad M, Shalviri M, Karbasi Z, Jafariazar Z, Asgarian R (2012). Evalution of antibacterial activity of three Iranian medical plants. African Journal of Microbiology Research.

[12] Cock IE (2008). Antibacterial Activity of Selected Australian Native Plant Extracts. The Internet Journal of Microbiology.

[13] Kittika N, Natta L, Orapian K (2007). Antibacterial Effect of Five Zingiberaceae Essential Oils. Molecules.

[14] Indh MN, Hatha AM, Abirosh C, Harsha U, Vivekanandan G (2006). Antimicrobial activity of some of the south-Indian spices against serotypes of Escherichia coli, Salmonella, Listeria monocytogenes and Aeromonas hydrophila. Brazilian Jornal of Microbiology.

[15] Dadgar T, Asmar M, Mazandarani M, Bayat H, Moradi A, Bazori M, Gaemi E (2006). Antibacterial activity of certain Iranian medical plants against methicillin resistant and sensitive Staphylococcus aureus. Asian Jornal Plant Sci.

[16] Davise FS, Albrigo LG, Citrus AB (1994). Citrus.

